# The trickle-down of political and economic control: On the organizational suppression of environmental scientists in government science

**DOI:** 10.1177/03063127221093397

**Published:** 2022-05-05

**Authors:** Sampsa Saikkonen, Esa Väliverronen

**Affiliations:** University of Helsinki, Helsinki, Finland

**Keywords:** suppression of scientists, power, control, organization, bureaucracy, expertise, public communication, freedom of expression, environmental research

## Abstract

In many countries, attempts to suppress scientists as public experts have become more prevalent. In democratic countries, environmental scientists have been a particular focus of control. This article looks at structures and mechanisms of suppression of government researchers. It is based on a qualitative analysis of ten in-depth interviews with environmental researchers being employed or engaged in government science. The analysis is influenced by a power-theoretical perspective on the suppression of science. By analyzing the interviewees’ accounts, it scrutinizes the different ways in which political and economic control can trickle down in research organizations such as state research institutes and come to affect individual researchers. The focus is especially on the interlinking of political and economic influence of external actors with different forms and practices of control at the organizational level. Three forms of such trickle-down are identified and discussed: internalization of political and economic control, external influencing and bureaucratic control, and economic/interest group influence in research organizations. We argue that these forms of control function as a filtering layer of suppression between political and economic control and individual scientists out of the public eye regarding government science.

There are strong political and economic interests around environmental research and the application of research data, so it is not surprising that environmental researchers are prone to pressure. There may be a straightforward use of political power by the government or lobbying by energy companies or think tanks. Types of suppression include defamation, false accusations, lawsuits, unjustified claims for scientific misconduct etc. According to Kuehn (2004: 368), universities, research organizations and scientific associations have limited power to defend researchers and their rights against suppression.

The years of President George W Bush (2001–2009) represented a new era when particularly environmental research was under attack (Cole, 2005, 2017; Resnik, 2008; Shulman, 2008).

For example, the well-known climate scientist James Hansen accused his employer NASA of violations of freedom of expression and censorship. According to Hansen, his e-mail was monitored, his public writing was required to be pre-screened and public appearances were watched (e.g. Rich and Merrick, 2007).

In Canada, during Stephen Harper’s government (2006–2015), environmental researchers faced similar problems. The government no longer wanted to commit itself to the Kyoto climate objectives and sought to promote Canadian industry by streamlining environmental legislation. The focus of the National Research Council’s funding was transferred from basic research to applied research that served to develop industry and innovations (Amend and Barney, 2016). At the same time, the freedom of expression of those working in state research institutions was significantly restricted. In 2007, research institutes received new communication guidelines that required researchers to ask for permission from their supervisors before they could contact the media or publicize their research. The researchers then no longer had the right to give interviews to journalists freely, and the communications managers of the research institutes became sensors (Magnuson-Ford and Gibbs, 2014).

In Finland, a similar, although more limited, case became public in 2010. A number of researchers working in the Finnish state research institute the Technological Research Centre (VTT) accused the leadership of the institute of silencing its researchers. One researcher working in VTT received a warning from his employer after criticizing nuclear power research just before the parliamentary nuclear vote. Another researcher was forbidden to send an opinion piece dealing with the use of peat in energy production to the biggest daily, *Helsingin Sanomat*. Researchers who appeared anonymously in the media also reported pressure and verbal abuse in situations where the findings and interpretations of the researchers differed from the VTT management’s energy policy guidelines. Some researchers had also been forbidden to appear as experts in parliamentary committee hearings. The dispute was raised by researchers in a context in which the government had just introduced its ‘green tax reform’, which would tax energy sources according to their emissions.

The Parliamentary Ombudsman took up the matter for investigation and commented on the violation of the freedom of expression of the researchers in 2011. According to the Ombudsman, ‘freedom of expression is also a matter for the official and the employee of a state institution’. The Ombudsman pointed out that VTT researchers have ‘freedom of science and research protected by the Constitution’. However, it seems that researchers working at VTT continue to face limitations to their freedom of expression (Väliverronen and Saikkonen, 2021).

This article focuses on how political and economic control can trickle down in research organizations such as state research institutes and come to affect individual researchers. We base our findings on an analysis of interviews on suppression with ten environmental scientists, who are employed or engaged in government science.

## Government science and Finnish state research institutes

While there is no simple definition of government science, due to its heterogeneity in different contexts and countries, it is commonly distinguished from academic science in being more directly tied to the government and the support of policy-making in the public interest (e.g. Irwin et al., 1997; Jasanoff, 1990; Klenk and Hickey, 2011; Resnik, 2008). Government science also differs from university research in being more conditioned by the practical demands of time and politics (Klenk and Hickey, 2011). Considered at the global level, government science is not only carried out at specific, dedicated institutional sites, but at various kinds of sites; it can also involve collaboration with universities, industry, the private sector or other actors (Irwin et al., 1997; Klenk and Hickey, 2011). The institutional culture of government science also varies between countries; for example, in Europe it tends to have affinities with academic science (e.g. Irwin et al., 1997).

In Finland, as in a number of other countries, government science is mostly organized and conducted in specific state research institutes, which historically have had important roles in the research and innovation system in Finland, producing knowledge and expert advice, and through these aiding policy-making but also societal and industrial development more generally (Late, 2014; Vähä-Savo, 2016).

The Finnish state research institutes are organized and operate under different ministries, conducting research and providing expert advice (Late, 2014). Currently, there are twelve state research institutes in Finland (Official Statistics of Finland, 2020). Of these, environmental research is conducted and related expert advice is provided especially by the Finnish Environment Institute, but also by the Finnish Meteorological Institute (climate and the environment), the Natural Resources Institute Finland (natural resources and the environment), the National Institute for Health and Welfare (environmental health), and VTT Technical Research Centre of Finland (e.g. energy, technologies, and the environment).

Finnish state research institutes also have a notable academic orientation in that there is also a focus on research excellence, and an aim that the institutes also produce high-quality research and predict future research (Late, 2014; OECD, 2011). The researchers working in the institutes therefore also produce academic publications, often expected by the institutes as employers; research collaboration with universities and researcher mobility between state research institutes and universities is also not uncommon (Eskola and Kirsilä, 2017; Late, 2014). Scientists working in Finnish state research institutes have been recognized as sources of expertise for policy-makers, but also more broadly in society, taking part in public discussions, for example by writing articles and commentaries to media (Late, 2014; Vähä-Savo, 2016). They have also commonly been free to do so in their areas of expertise, even in a critical tone, without facing interference (e.g. Karvonen, 2011).

In the past 15 years, and especially after an advisory board was established in 2006–2007 for developing the research conducted in state research institutes, the Finnish state research institutes have been under considerable structural reform and reorganization (Heikkilä, 2007; Late, 2014). The number of the institutes has been systematically reduced by merging institutes with each other and with universities, and in 2015–2016 over 60 million euros was cut from the budgets of the state research institutes and allocated to new, competitive research funding instruments, with 52.5 million euros being allocated to the new and more tightly politically steered Strategic Research Council that allows funding to certain focus and thematic areas it defines each year (Late, 2014; Tammi, 2016). These structural changes have amounted to increased political steering of government science and state research institutes, which has affected, through increasing control, the research conducted in, knowledge provided by, and the culture within government science and state research institutes as organizations (e.g. Heikkilä, 2007; Kirsilä, 2016; Tammi, 2016). Moreover, attempts to restrict or suppress the employed scientists’ freedom of expression as experts have also emerged, as the VTT case demonstrates (Karvonen, 2011; Väliverronen and Saikkonen, 2021).

## Analytical framework: A system of power perspective and the organizational suppression of scientists

Direct censorship is a visible form of suppression (Delborne, 2016; Martin, 1999, 2001). In general, direct censorship is typically practiced by the state (e.g. Post, 1998). However, much of the suppression of scientists can be more subtle, distributed and patterned. Martin (1999: 109) defines a *system of power* in terms of:

a set of patterned social relationships, usually reaffirmed but sometimes challenged by the behavior of individuals, which provides differential opportunities to groups and individuals to influence the behavior of others. A system of power in this usage is compatible with a nonreified interpretation of social structure; it is intended to refer both to power associated with social structure and to power exercised on a local scale, for example between individuals within an organization.

It is important to scrutinize how *influence* is exerted and power is practiced between groups and individuals. Those who aim and strive to suppress scientists from communicating findings or views are not necessarily themselves engaged in any direct contact with the scientists. Rather, suppression attempts can function through influence exerted indirectly. It is therefore important to pay attention to how power functions and flows in suppression attempts. Different power resources can exert influence, and influence can take forms that vary from coercion to inducement or persuasion (Martin, 1999; Scott, 2001; Wrong, [1979] 2017). Building on Delborne’s (2008) notion of scientific dissent as a practice (rather than as a position), in this study we examine *practices* involved in the organizational suppression of scientists.

To analyze how power is exercised in science, a number of scholars in science and technology studies (STS) have emphasized the importance of organizational aspects and dynamics and how these condition the work and activities of scientists (e.g. Blume, 1974; Frickel and Moore, 2006; Martin, 1981, 1999). One strand of research within STS has looked at the production of ignorance, in which the interplay of external influence and internal and organizational dynamics of science has also been noted, although the focus tends to be on the dynamics of the social production and sustaining of ignorance (e.g. Jeon, 2019; Pinto, 2015; Richter et al., 2018). Yet empirical studies and theorizing regarding the organizational dynamics of *suppression* of scientists specifically remain notably scarce in the existing STS literature (Martin, 1999).

Proceeding from a system of power perspective on suppression, this article explores the organizational dynamics of suppression in the context of government science. Our focus is specifically on the different ways in which political and economic control by external actors can trickle down in research organizations and affect individual researchers. We analyze interviews with ten environmental scientists employed or engaged in government science.

## Data and methodology

The ten environmental scientists were chosen to be interviewed on the basis that they had experience of being employed or otherwise engaged in government science, and that they could provide a view on influencing and suppression based on their experience of having faced such attempts. The interviewed scientists had experience of doing research in a variety of the Finnish state research institutes. In addition, some of the interviewees also had other, advisory type of experience of engaging in government science, such as experience of serving in advisory panels at the science–policy interface. Four of the interviewees held positions as senior or principal scientists and six were professors or research professors. Their research orientation and background varied and was in physics and energy research (environmental and climate impacts), marine science and environment, social sciences (environmental policy), economics (environmental, climate, and natural resource economics), environmental health, and forest sciences. They had extensive experience as environmental researchers, ranging from about 20 years to almost 40 years of experience, with an average research experience of 27.4 years.

We conducted our interviews with these ten scientists between December 2018 and February 2019. The semi-structured, in-depth interviews lasted from 45 to 85 minutes, and were recorded and transcribed for analysis. The scientists were queried about: the societal atmosphere for discussing environmental issues as an expert, experiences and descriptions of suppression attempts and their consequences, the role of the research organizations in connection with researchers’ activities as public experts and instances of being subjected to suppression, the roles of colleagues, and whether and how being subjected to suppression relates to conducting and making choices in research. The interviewees proceeded in a conversational manner and by allowing the issues queried about to be explored freely and in-depth by the interviewees (Longhurst, 2009).

We applied qualitative content analysis to identify themes and patterns (Hsieh and Shannon, 2005; Silverman, 2015). First, we analyzed and classified the data based on what kind of social actors and control the interviewees’ noted. Five codes were derived and used: (1) top-down control of political actors and corporate stakeholders, (2) control practiced in the research organization, (3) outside influence by interest groups and economic actors, (4) control practiced by colleagues and other researchers, and (5) citizens and their feedback. If two or more of these emerged in the accounts simultaneously, multiple codes were used accordingly in the coding.

We then extracted and attended to more closely the parts of the interviewees’ accounts in which the kinds of overlapping described above occurred. In this phase, we scrutinized interviewees’ descriptions of the practices, motives, dynamics between actors, and consequences as they accounted for their experiences of suppression and silencing. We attended to how the interviewees described these in terms of how external control links with control and its practices in research organizations. Based on the analysis we identified three different forms of the trickling down of political and economic control in research organizations: internalization of political and economic control, external influence and bureaucratic control, and economic or interest group influence.

## Analysis

### Internalization of political and economic control

Political and economic control can take the form of organizational internalization of expectations, to such an extent that researchers’ public statements perceived to deviate from or counter such expectations can become viewed as problematic. In practice, this form of political and economic control is interlocked with organizational control, having centrally to do with how managers internalize expectations from ministries or corporate stakeholders:

We had drafted a letter to the editor as researchers … which our manager turned down, namely, that you cannot send this [to the media]. And we [the researchers] thought that this was a miscarriage of justice … it felt really wretched, especially as we did not understand the grounds for this. It [the letter to the editor] was a statement to an ongoing discussion, and even rather solution-centered, in which we provided recommendations for action. And then we got to know … that [the grounds for the decision was that] this endangers our certain funding arrangements. And it took a really long time for me, and I don’t know if I have even still recovered from this properly, that I should, as a servant of tax-payers, be thinking whether some stakeholder becomes offended by some letter to the editor to the extent that they will not finance our research.

Concern about funding is a likely concern in government science organizations, which crucially depend on funding allocations. Public communication that is perceived to contradict governmental environmental and energy agendas can be discouraged or suppressed by managers wanting to avoid cutbacks or even withdrawal of funding. However, some of the interviewees also brought up how, in projects involving corporate stakeholders, managers can attempt to block public communication by researchers in which, for example, critical findings regarding the environmental impacts of an energy source or technology studied are addressed. Such attempts can stem from the managements’ fear of losing corporate stakeholders as economically resourceful partners in current and future projects, as illustrated in this account by an experienced research professor:

[Research institute x]^1^ regards itself being foremost, or very much, in support of industry interests. … and then, if those [the employed researchers’ public writings] were of the type that they were in a way against some company’s interest, then a reprimand could follow, of the sort that … one should not write such public writings … and that can consequently also cause harm to the [research institute x]. The first issue in the context of which this emerged and occurred was the issue of peat burning [to produce energy]. … and I normally did not ask [permission] when I wrote a piece … I just wrote. So, the reprimands came afterwards then. But some did ask the superiors whether they can write. And the superiors forbid it, or I don’t remember whether they utilized an absolute ban, but nevertheless in some way clearly indicated that it is not good to write.

An institutional culture of preparedness can develop in which the researchers’ public activities and communications are more constantly monitored in the light of specific expectations. This was noted to be typical, especially regarding environmental issues that have high policy relevance, as the quote below illustrates:

I know that in state research institutes, especially maybe top management, they are heedful with respect to what is thought in the ministries. … I also know that, a certain topic, that is related to these effects on climate by the additional use of forests, so for example a person who knows much about this topic, who is employed by [research institute x], is him/herself very careful about what to say or write about this topic, if the saying or writing is maybe, if it has a very clear statement to this topic, and [they] want to check it for example with the superior. I know that there they think about these things automatically in this way.

Some of the interviewees also said that not only the management internalizes external economic and political expectations, but that environmental researchers do so, too. Such more pervasive internalization of political and economic control can therefore cause the researchers to be more constantly cautious and keep expectations in mind. They might refrain from challenging the central agendas of political and economic stakeholders to avoid causing trouble for themselves and, collectively, other researchers employed in the organization. This is illustrated in the account below, regarding a situation in which the state research institute, where the interviewed professor was employed for over a decade doing forest-related research:

And once, twice a year the management always indicated that our staff would be reduced. This pressure regarding reductions and possible layoffs of staff was present all the time. At the same time, there was this political pressure regarding the right politics of forestry, and should public grants still be directed towards the forest sector. And in this kind of situation the staff starts to have a certain feeling on what is expected from us. That if we now cannot carry out those expectations our existence and the pressure of reduction directed at the staff is greater than before. … And then, if some researcher criticizes some established way of thinking, which is politically correct and favoured by largest interest groups. That is not in such a situation regarded [by the institutes’ staff] as desirable at all, rather it would be good that everybody stay in line, and no-one causes trouble.

The internalization of political and economic control in research organizations constitutes a subtle but pervasive form of the trickling down of political and economic control. As the interviewees’ accounts analyzed here make explicit, especially those in managing positions in research organizations can readily internalize external political and economic expectations of ministries or corporate stakeholders, and discourage or more actively suppress public communications that they perceive to contradict these. However, as highlighted above, political and economic expectations can also become more pervasively internalized within research organizations, and affect the practices and mindset of the researchers regarding what and how they can communicate as public experts (Väliverronen, 2021). Fear of causing economic risk for the research organization and for those employed can drive both such forms of internalization of political and economic control.

### External influencing and bureaucratic control

The influence of politically and economically powerful external actors can become forcefully interlocked and work through hierarchical, bureaucratic control in the research organization. In contrast to the internalization of political and economic control, this form involves an active attempt by external actors to apply pressure to actors high enough in the research organization, such as managers, who can exert bureaucratic control over researchers by directing and sanctioning them. Influencing research organizations to exert bureaucratic control over researchers often constitutes a powerful form of silencing:

Then the most concrete way in which [research institute x] and the actors in the background can influence something like this has notably to do with [exerting influence over] the advancement of a researcher’s career. Like if one writes too negatively, according to some involved party, then after this one’s career as a researcher stops, or one can even be laid off.

There are various punitive forms of bureaucratic control that can be exploited to suppress employed researchers’ activities and communications. These forms range from warnings and reprimands to transfers to other units, denials of career advancement, threats of redundancy and actual layoffs (see Kuehn, 2004; Martin, 1999).

Instances of suppression through bureaucratic control can remain out of the public eye, unless researchers subjected to them make them publicly known. For many researchers, becoming a whistleblower can, however, bring on further risks.

Management at research institutes can easily be contacted by external political and economic actors – often by phone, and thus in ways difficult to trace. This was reported to result in the researchers in question being questioned and reprimanded by the manager who had been contacted:

Like at the times at [research institute x] the action was of the sort that if the researchers said something [unwanted by external actors] in the media, then the managers were phoned straight away, with the intent of questioning this. So, it [the pressuring] is indirect, it has not been directed straight at me, but to those in managing positions. And indeed, and this is now maybe the most essential, that [person x] when being in [a leading position in ministry x], had in one event … in which there were lots of executives and other actors from industry and big corporations present – so influential people and also my [research institute x’s] line manager of that time from [unit x] – stated that … these studies of [researcher x, talks about him/herself] are extremely harmful with respect to the promotion of interests of Finland. So that sort of case. And then my manager called me and told me about this. And then we went through this conversation about what should now be done. And I remember asking if one should start lying about these research results. So, this was quite … like when it [the pressuring] comes from [a person in leading position] from the ministry directing us, that is quite …

This way of exerting influence, by directly contacting the researchers’ employer and demanding them to take action (e.g. Hoepner, 2017; Martin, 1999) is immediate and takes place out of the public eye. But it is notably also a tactic that depends on establishing close relations with individuals who hold bureaucratic power, typically managers, so that there is a high degree of certainty of action. Without those close relations, direct contact runs the risk of backfiring (Martin, 2007).

Stakeholders can use their roles in large-scale joint projects – in committees, working groups, workshops, and other collective steering mechanisms – not merely as sites of information exchange and deliberation but also as sites for exerting control over researchers regarding desirable outcomes and how these should be communicated:

These are maybe more challenging, these kind of projects where there is indeed both public and industrial money involved … that we also did when [I was] at [research institute x]. Like they were quite something, those conversations, and I do remember these kind of workshops, which were also held a myriad of times. And in a way it was somehow that kind of a process that the forestry industry put in lots of money. Like we had some three-year project. They had clear expectations that now they get that kind of results that they can utilize to market like how awesome the Finnish forest industry is from the climate perspective. And then, when they saw that, well, these results won’t go on the track which they had hoped for … these workshops were held. They were held countlessly. And always if they had some arguments, then those were grasped and systematically analyzed, and then after this process we returned to the starting point there at the end, so one could conclude that ho-hum, nothing had progressed. But they were pretty strict.

In such joint projects, forcefully arguing, imposing views and vetoing or presenting demands in frequent collective meetings can allow political actors and corporate stakeholders to control researchers and suppress unwanted lines of inquiry and communications.

As more bureaucratically structured research organizations commonly do public communication of research as a separate practice, and often also have separate personnel or department for this purpose, one way by which external political and economic actors influence and suppress public communication is to exert pressure over those who are responsible for, or are able to control, the public communications and relations of the organization:

… if we take, say, [corporation x] as an example, so if some peat-criticizing writing is published somewhere, then this representative surely will, if this representative knows the involved researchers’ bosses’ boss, contact [them] and say, that you made this public in this sort of way, that could you have done the press release slightly differently. … This is one, important part of the use of power these press releases. … So, if the researcher’s results cannot be controlled, then the press releases can be. … And this also doesn’t show then. Of course, the press release goes in most cases to the researcher to be read [checked before sending], but the researcher is busy … so the researcher might accept sorts of softening and exclusion of certain things, and things like that.

External actors can therefore exploit the bureaucratic structures to suppress the public communication of unwanted findings or views.

Some interviewees also emphasized that attempts by political and economic actors to suppress research can be resisted. Clear codes of conduct can be established that set out how and on what grounds managers or others can intervene in the activities and communications of researchers, as brought up by a senior researcher:

Well, if this kind of code of conduct did not exist, in which it is clearly defined what is permitted and what is not. As long as there is a grey zone, political control is easier to use, or the control is easier to use. … if there is a specific code of conduct, then you can always question it more easily, like that okay … it is illegal to make such a restriction. But if this is unclear … then you must fear.

Success in getting such codes of conduct established in one state research institute can, then, also become known and referred to by researchers in other institutes. In this way, the codes of conduct can therefore potentially come to spread and get applied also outside the research organization within which they were first established:

And of course it is good, that as [research institute x] … has established this kind of code of conduct, then an employee in some other state research institute certainly gets to know through some colleague our code of conduct and can say, that there they have this kind of code of conduct in place ….

The exertion of political and economic influence to control and suppress researchers through persons with bureaucratic power and bureaucratic practices described in this section constitutes a core mechanism of political and economic control of state research. Such indirect suppression can, at its most extreme, involve pressure to impose highly punitive bureaucratic measures. However, there are also forms of influence through bureaucratic control and practices that involve less punitive measures, but that can also be effective at suppressing findings and views.

### Economic or interest group influence in research organizations

Interviewees identified a third way in which political and economic control can trickle down. It had to with how external interest groups and economic actors can coax and nudge individual researchers to adopt their views and interests, which might in turn affect the actions and statements of other researchers. This coaxing can take place even before researchers present undesirable findings and statements in public. In Finland, economic interest groups were seen as exerting their influence in this way, especially because state research institutes are linked to the policy world:

… it is astonishing even in this day’s Finland … the interest group control or something like that. Some unbelievable stories colleagues tell about how it works. Then some researchers are in favor of interest groups, and others are not. … it is made known, that you will have it nicer when you are on our side, and something like this. Like make your life easy. … I know that there is all kinds of networking … like getting invited to pre-Christmas parties and conversations, and I know that this clearly happens. We even know some of our researchers, who are among some interest group’s insiders. Within our own institute. And this is maybe related to this kind of [control] between colleagues, like when we get to more difficult areas, then remarks can well be made, that you can’t say that. … It is like this kind of peer control. Like those researchers are peers and sometimes we are all well chummy, and like this. So it varies a little, but these things exist.

Central to this way of exerting influence is therefore that interest groups and economic actors gain influence among researchers who then can oppose and argue against their colleagues. As the quote above highlights, this form of control demands specific and possibly continuous efforts to gain influence over some researchers. These researchers can, then, mediate the influence of the interest group or economic actor, and to exert peer control. Such horizontal control is not mutually exclusive with either of the other forms of trickle-down control, but forms a different axis.

The pressure from interest groups and economic actors is not only related to specific or single topical issues, but also to broader, paradigmatic issues within research fields. This was seen to hold especially regarding research and its communication on issues with both environmental and economic significance, such as forestry in the Finnish context:

… now if you think about Finland’s most important natural resources, and quite a bit of environmental issues are connected to woods in Finland, and there the majority of researchers, they have an applied ecology training or slightly also – some ecologists would say, that they are forest engineers, not ecologists. So they don’t have education in economics. … And I do have a very strong multidisciplinary orientation because this economics point of view is not credible in forestry, if the models do not have the natural sciences in order. … and then this basic principle constituting Finnish forest research is silviculture, in which real understanding of economics is quite scarce. And from this follows, that these results that I get from this [economics] standpoint, do not always match with this Finnish [silvicultural] approach. And when I participate in this way in this [public] discussion, these forestry researchers feel that I am stepping on their territory. … And when there have been these … sort of suppression type of activity [by peers], these have emerged mostly from this direction. And this group, then … they have a certain kind of relationship to interest groups, and the interest group pressure often gets channeled in a way through them then.

In cases where unwanted views gain publicity, there can be direct requests to discredit them, involving already-influenced researchers:

I do know that interest groups have also commissioned researchers to engage in this kind of debunking … the interest groups have contacted certain researchers and asked them to debunk such a person, who presents problematic results. So, this sort of thing also happens.

Notably, there is also a *corporatist* tendency in the exertion of influence horizontally. In this way, the interest groups or other economic actors can aim to extend and ensure the representation of their views and interests at the level where constitutive knowledge issues are debated.

## Conclusion

In this study, we investigated the suppression of government scientists by focusing on how political and economic control by external actors can trickle down in research organizations and affect individual scientists. Our approach and analysis was grounded in a system of power perspective (Martin, 1999).

Based on our analysis, we identified three forms of the trickling down of political and economic control. The first form involves the organizational internalization of governmental or corporate expectations and interests, so that these guide the scrutiny of the employed researchers’ expert statements. Statements contradicting such expectations and interests may be discouraged or otherwise suppressed. Managers at state research institutes may be particularly prone to internalizing political and economic control. The notion of the internalization of political and economic control here thus supports, but also extends, previous notions of the role of management regarding the suppression of scientists (e.g. Hoepner, 2017; Martin, 1981, 1999).

Suppression and self-censorship of scientists can be related (e.g. Delborne, 2016; Hoepner, 2017; Kempner, 2008; Martin, 1999, 2001), and the internalization of political and economic control within state research institutes can be perceived and conceptualized in terms of institutional self-censorship. Such self-censorship therefore constitutes an issue for the free communication of findings and views, in that political and economic expectations and interests come to guide considerations, or lead to overt carefulness, in the public communication of findings and expertise.

The second form, external influence and bureaucratic control, refers to the exertion of political and economic influence by external actors to suppress researchers by contacting persons with bureaucratic power in research organizations and by exploiting bureaucratic practices. In this way, political and economic control can trickle down in research organizations and affect individual researchers without any external actors themselves directly exerting pressure on individual researchers. Supporting previous observations, our findings indicate that such indirect influence can involve pressuring research organizations and their management to impose punitive bureaucratic measures over researchers (e.g. Kuehn, 2004; Martin, 1999). However, extending such observations, this study also shows how softer forms of influence by external actors, such as by exploiting collective steering mechanisms and exerting influence over the research organizations’ public relations, can also affect the public communication of findings and expertise. This illuminates the problematic role of PR in communicating science (Väliverronen, 2021).

The third form, economic or interest group influence of researchers, displays how external actors can aim to gain influence horizontally by establishing good relations with some researchers to attack and suppress other researchers’ findings. Moreover, the study points to corporatist tendencies involved in this third form of trickle-down control. These tendencies have not received much attention, but their scrutiny can help us to understand the interlinking of political, economic and organizational control of government science.

The forms of trickle-down control that we have identified here constitute and function as a *filtering layer* of suppression. Control can become filtered through organizational levels in more hierarchically structured research organizations, such as state research institutes. This advances our understanding of the suppression of scientists as an issue related to power between political and economic, organizational and individual levels. The findings of this study can therefore contribute to a better understanding of the current problems of public expertise and its silencing in society.

## Notes

1. [Research institute x] is used throughout this article as an anonymization marking in the interview quotes for all the Finnish state research institutes mentioned by name during the interviews, not just for a single, specific state research institute.
